# Antioxidant Extract from *Cleistocalyx nervosum* var. *paniala* Pulp Ameliorates Acetaminophen-Induced Acute Hepatotoxicity in Rats

**DOI:** 10.3390/molecules27020553

**Published:** 2022-01-16

**Authors:** Arpamas Chariyakornkul, Waristha Juengwiroj, Jetsada Ruangsuriya, Rawiwan Wongpoomchai

**Affiliations:** 1 Department of Biochemistry, Faculty of Medicine, Chiang Mai University, Chiang Mai 50200, Thailand; arpamas.ch@cmu.ac.th (A.C.); waristha_thus@hotmail.com (W.J.); jetsada.ruang@cmu.ac.th (J.R.); 2 Nutraceuticals and Functional Foods Research Cluster, Faculty of Medicine, Chiang Mai University, Chiang Mai 50200, Thailand

**Keywords:** acetaminophen, *Cleistocalyx nervosum* var. *paniala*, hepatotoxicity, oxidative stress

## Abstract

The indigenous purplish red fruit, *Cleistocalyx nervosum* var. *paniala* (CN), is grown in northern Thailand. The aqueous extract of CN pulp is known to exhibit antioxidant and anticarcinogenic properties. To search for an antioxidant fraction separated from CN, various hydroalcoholic extractions were performed. The acidified ethanolic extract of CN obtained from 0.5% (*v*/*v*) citric acid in 80% (*v*/*v*) ethanol yielded greater polyphenol content and DPPH radical scavenging activity when compared with other hydroethanolic extracts. Cyanidin-3-glucoside is a major anthocyanin present in the acidified ethanolic extract of CN (AECN). At a dose of 5000 mg/kg bw, an anthocyanin-rich extract was found to be safe when given to rats without any acute toxicity. To examine the hepatoprotective properties of AECN, an overdose of acetaminophen (APAP) was induced in a rat model, while silymarin was used as a standard reference. The administration of AECN at a dose of 300 mg/kg bw for 28 days improved hepatocyte architecture and modulated serum alanine aminotransferase levels in APAP-induced rats. Furthermore, it significantly decreased serum and hepatic malondialdehyde levels but increased hepatic glutathione content, as well as glutathione peroxidase and UDP-glucuronosyltransferase activities. In conclusion, AECN may effectively reduce oxidative stress induced acute hepatotoxicity in overdose APAP-treated rats through the suppression of oxidative stress and the enhancement of the antioxidant system in rat livers.

## 1. Introduction

Antioxidants are natural or synthetic compounds that help delay or prevent free-radical-induced biomolecule and cell damage. Antioxidants can be divided into the following two categories: enzymatic and non-enzymatic antioxidants [1]. The former category includes superoxide dismutase (SOD), catalase (CAT), glutathione peroxidase (GPx), and glutathione reductase (GR), while the latter is comprised of phytochemicals, such as β-carotene, lycopene, flavonoids, and vitamins, and certain metabolic molecules such as glutathione (GSH), coenzyme Q10, and bilirubin [1,2]. Antioxidants prevent oxidative stress via the neutralization of reactive radical species including reactive oxygen species (ROS) and reactive nitrogen species (RNS). These are the key molecules underlying a number of chronic diseases such as cancer, cardiovascular diseases, and neurodegenerative diseases [2,3]. The imbalance between antioxidant and free radical generation systems can lead to the accumulation of defective macromolecules such as proteins, lipids, and nucleic acids, along with the alteration of enzymatic activity, cellular signaling, vital protein functions, and oxidative stress [2,3].

The liver, a key organ involved in xenobiotic metabolism, can help to either biotransform or detoxify drugs, alcohol, certain toxicants, or other chemical substances due to the presence of a number of detoxifying enzymes. Reactive metabolites and ROS are generated from the metabolism of the xenobiotics, especially during phase I metabolism. Reactive metabolites and ROS are known to induce liver injury and malfunction by the covalent modification of hepatic biomolecules, such as proteins, DNA, lipids, and membranes. This can potentially lead to mitochondrial dysfunction, oxidative stress, and endoplasmic reticulum (ER) stress [4,5]. Drug-induced liver injury is a common cause of acute liver failure and chronic forms of hepatotoxicity [6], particularly acetaminophen (APAP). Although APAP is considered safe in terms of its antipyretic and analgesic effects when administered in an approved therapeutic dose, overdosing can result in hepatotoxicity and liver failure. APAP is metabolized by cytochrome P450 isozyme 2E1 to produce hazardous intermediate ROS, which is known to cause hepatotoxicity [5,7].

A large group of natural antioxidants that are present in vegetables and fruits have been reported for their biological activities under oxidative conditions. They are able to scavenge free radicals, increase antioxidant enzyme activities, induce apoptosis, and increase antioxidant capacity in serum [8,9]. Some flavonoids obtained from plants can directly bind with various types of ROS, including hydroxyl radicals (^•^OH), superoxide radicals (O_2_^•^^−^), and hydroperoxides of linoleic acid (LOO^•^) [10,11]. The leaf extracts of *Vitex doniana* and *Mucuna pruriens* can improve the antioxidant system involved with carbon tetrachloride-induced oxidative stress in rats by decreasing liver MDA levels whilst statistically increasing SOD and CAT activities [12]. The hepatoprotective effects on APAP-induced liver damage are indicated by an improvement in GSH contents and an inhibition of lipid peroxidation, while the enhancement of hepatic antioxidant enzymes has also been reported in certain polyphenols. These include hesperidin [13] and apigenin [14] derived from oranges and grapefruits, gallic acid [15] and chlorogenic acid [16] obtained from a number of plants, and anthocyanins derived from purple sweet potatoes [17].

*Cleistocalyx nervosum* var. *paniala*, known as Ma-kiang, is an edible purplish fruit that is commonly cultivated in northern Thailand. Previous studies have demonstrated that the aqueous extract of *C. nervosum* pulp can produce an antimutagenic effect against mutagen-induced mutagenesis in *Salmonella* Typhimurium strains TA98 and TA100 [18]. Moreover, this fruit has exhibited chemopreventive effects on the early stages of hepatocarcinogenesis in rats by reducing lipid peroxidation and enhancing antioxidant enzymes [19]. Several studies have shown that the type of solvents employed for phytochemical extraction can influence the antioxidant activity of plants [20]. To search for a safe antioxidant rich fraction of *C. nervosum* pulp, various hydroethanolic extracts were used to verify their safety and to further investigate their effectiveness using standard acute oral toxicity, while also exploring their toxicity in rats and the hepatoprotective mechanism involved with overdose APAP-induced oxidative stress.

## 2. Results

### 2.1. Phytochemical Content and Antioxidant Capacity of CN Pulp Extracts

The phytochemical contents of CN pulp extracted from different ethanol concentrations ranged from 0 to 95% and are shown in Table 1. The aqueous extract revealed the lowest chemical contents, whereas the 80% (*v*/*v*) ethanol extract yielded the highest number of chemical constituents. Total phenolic compounds, flavonoids, and anthocyanins were found to vary depending upon the ethanol concentrations that ranged from 20 to 80%. However, when the ethanol concentration reached 95%, the amount of these phytochemicals was lower than 80% ethanol extraction. To increase the efficiency of the extraction, 0.5% (*v*/*v*) citric acid was added into 80% (*v*/*v*) ethanol. The results indicated that the contents of total phenolics, flavonoids, and anthocyanin compounds of the acidified ethanol extract (0.5% (*v*/*v*) citric acid in 80% ethanol) were greater than those of the ethanol extracts. Values of 50.47 ± 4.67 mg eq GAE/g extract and 7.28 ± 0.03 mg eq C3G/g extract of total phenolic and anthocyanin compounds were recorded, respectively. Remarkably, the acidified ethanol extract presented the greatest antioxidant activity. Among the non-acidified ethanolic extracts, 60–95% of the ethanol extracts provided high DPPH scavenging capacities. The antioxidant potency of all the extracts declined in the following order: acidified ethanol, 60–95% ethanol, 40% ethanol, 20% ethanol, and aqueous extract. The IC_50_ value of the acidified ethanol extract was 3.25 ± 0.27 mg/mL, while ascorbic acid, a standard antioxidant, was recorded at 0.18 ± 0.08 mg/mL. The acidified ethanol extract of CN pulp (AECN) was selected for further investigation.

**Figure 1:**
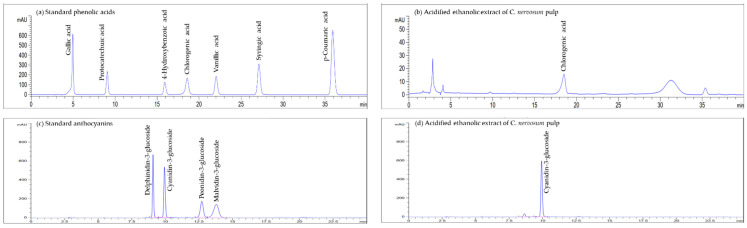
HPLC chromatograms of (**a**) mixtures of standard phenolic acids, (**b**) phenolic acids in acidified ethanolic extract of *C. nervosum* pulp, (**c**) mixtures of standard anthocyanins, and (**d**) anthocyanins in acidified ethanolic extract of *C. nervosum* pulp.

The phytochemical content, including certain phenolic acids and anthocyanins in AECN, were determined using HPLC. The main phenolic acid present in AECN was chlorogenic acid (0.88 mg/g extract). Moreover, the amount of C3G in AECN was 28.29 mg/g extract, which was the major anthocyanin in this extract. Accordingly, the chromatograms of AECN are presented in Figure 1.

### 2.2. Acute Toxicity Test of AECN Extract

The rats receiving 5000 mg/kg bw of the AECN extract were found to be healthy and showed no clinical signs of toxicity throughout the observational period. All the treated rats survived until the end of the experiment (14 days). The treated groups showed no significant differences in their average body weight in terms of the consumption of food and water when compared to the control rats. The relative organ weights were not significantly different when compared with the controls (data not shown). It can be suggested that AECN was safe, while the value of LD50 was greater than 5000 mg/kg bw.

### 2.3. Effect of AECN on Serum ALT Level and Oxidative Stess Markers in APAP-Induced Hepatotoxicity

**Figure 2:**
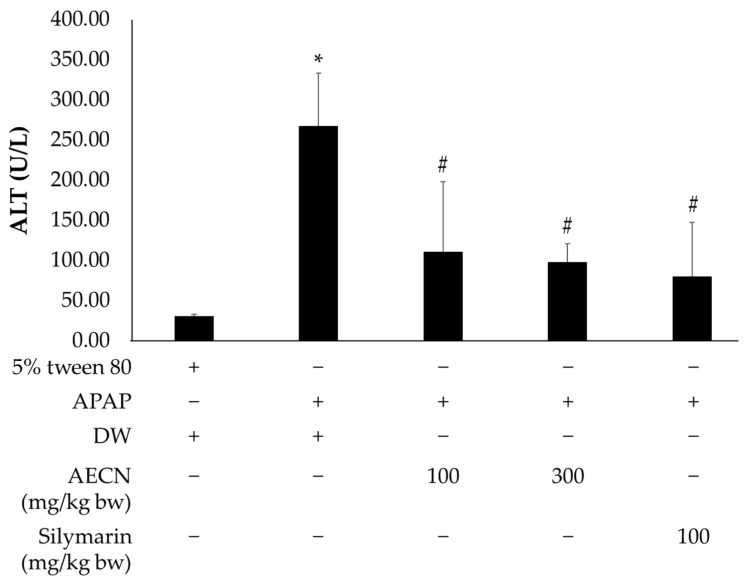
Effect of AECN on serum ALT in APAP-treated rats. Data are expressed as mean ± SD values (*n* = 8). * Significantly different from the control group (*p* < 0.05), # Significantly different from the APAP-treated group (*p* < 0.05). AECN: acidified ethanolic extract of *C. nervosum* pulp; APAP: acetaminophen at dose 3000 mg/kg bw; DW: distilled water used as a solvent control for AECN; 5% tween 80 used as a solvent control for APAP.

The level of serum alanine aminotransferase (ALT) was used as a biochemical marker for the evaluation of early hepatic injury. The administration of APAP was found to cause liver injury, as evidenced by an increase in serum ALT when compared with the control rats. Meanwhile, a pretreatment of 100 and 300 mg/kg bw of AECN effectively reduced the APAP-induced elevations of serum ALT. The administration of 100 mg/kg bw of silymarin, a known herbal antioxidant, also substantially prevented liver injuries. Accordingly, the results are presented in Figure 2. Table 2 illustrates the serum and liver MDA content in both the control and experimental rats. The level of serum and hepatic MDA in APAP-treated rats significantly increased when compared to the control group. The pretreatment of 100 mg/kg bw of AECN significantly decreased the serum MDA content. Conversely, the rats administrated with 300 mg/kg bw of AECN and silymarin exhibited statistically lower serum and hepatic MDA levels in comparison to the group that was treated with APAP alone. However, a 100 mg/kg bw dose of AECN had no effect on the MDA levels on the liver when compared with the APAP-treated group.

Remarkably, there was no change in the serum GSH content in all the treatment groups. In comparison to the control group, APAP dramatically depleted the hepatic GSH content. Pretreatment with a high dose of AECN reversed hepatic GSH depletion. Furthermore, the rats given a low dose of AECN and silymarin did not exhibit an improved degree of GSH depletion. The results are presented in Table 2.

### 2.4. Effect of AECN on Hepatic Histopathology in APAP-Induced Hepatotoxicity

**Figure 3:**
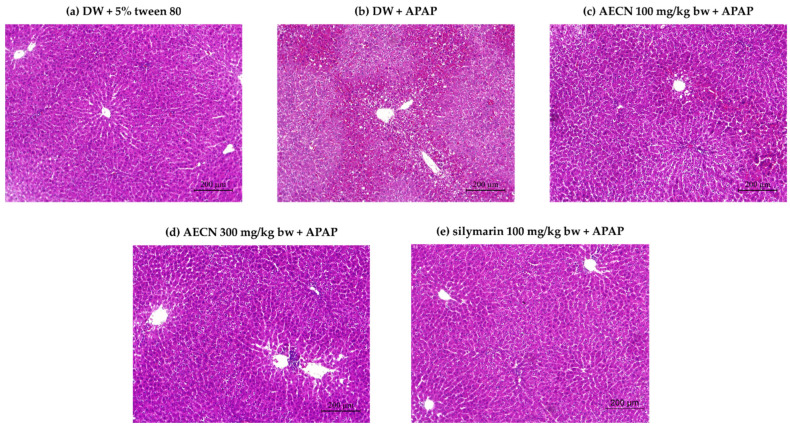
Histopathological analysis of liver sections of rats (10×). DW + 5% tween 80 (**a**), DW + APAP (**b**), AECN 100 mg/kg bw + APAP (**c**), AECN 300 mg/kg bw + APAP (**d**), and silymarin 100 mg/kg bw + APAP (**e**). AECN: acidified ethanolic extract of *C. nervosum* pulp; APAP: acetaminophen at a dose of 3000 mg/kg bw; DW: distilled water used as a solvent control for AECN; 5% tween 80 used as a solvent control for APAP.

The normal architecture of liver tissues among the control rat livers included hepatocytes, central veins, sinusoids, and portal tracts (Figure 3a). As has been demonstrated in Figure 3b, the livers of APAP-induced rats showed severe hepatocellular injury, including inflammatory cell infiltration, hepatic necrosis, sinusoidal congestion, and nuclear alterations. The existence of centrilobular coagulative necrosis, as determined by cell detail loss, was found in these livers along with some histologically intact hepatic parenchyma. The rats pretreated with 100 and 300 mg/kg bw of AECN and 100 mg/kg bw of silymarin were found to be free from liver damage initiated by APAP. These results are illustrated in Figure 3c–e, respectively. The absence of necrosis, normal hepatocytes, and normal architecture in AECN and silymarin pretreatments was observed.

### 2.5. Effect of AECN on Antioxidant Enzyme Activities in APAP-Induced Hepatotoxicity

**Figure 4:**
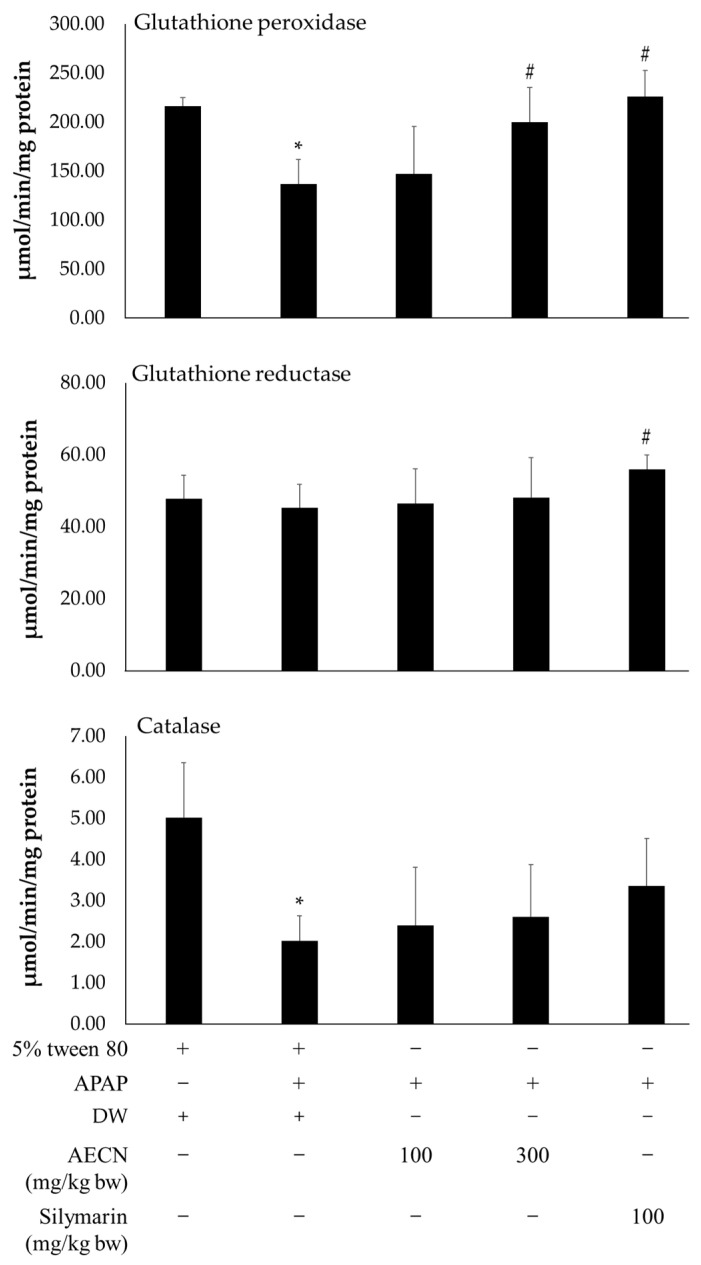
Effect of AECN on hepatic antioxidant system in APAP-treated rats. Data are expressed as mean ± SD values (*n* = 8). * Significantly different from the control group (*p* < 0.05), # Significantly different from the APAP-treated group (*p* < 0.05). AECN: acidified ethanolic extract of *C. nervosum* pulp; APAP: acetaminophen at a dose of 3000 mg/kg bw; DW: distilled water used as a vehicle for AECN; 5% tween 80 used as a solvent control for APAP.

Hepatic antioxidant enzyme activities, including GPx, GR, and CAT, were measured and are shown in Figure 4. Hepatic GPx activity was dramatically diminished in the APAP-treated group when compared to the control group. The APAP-induced depletion of hepatic GPx activity was alleviated by 300 mg/kg bw of AECN and 100 mg/kg bw of silymarin but not by 100 mg/kg bw of AECN. The treatment of AECN at 100 mg/kg bw did not show any changes in GPx activity when compared with the APAP-treated group.

The treatment of APAP had no effect on the GR activity when compared to the control group. Similarly, alterations in GR activity were not observed in all the treatments involving AECN. On the other hand, pretreatment with silymarin showed significant increases in GR activity when compared with the APAP-fed rats.

The group treated with APAP alone exhibited a statistical decrease in CAT activity when compared with the control group. There was no difference in CAT activity in the comparisons made between the group pre-treated with AECN at all doses and the group treated with APAP alone. Furthermore, the activity of CAT in the silymarin-administrated rats was not altered when compared to the APAP-initiated rats. 

### 2.6. Effect of AECN on Xenobiotic Metabolizing Enzyme Activities in APAP-Induced Hepatotoxicity

**Figure 5:**
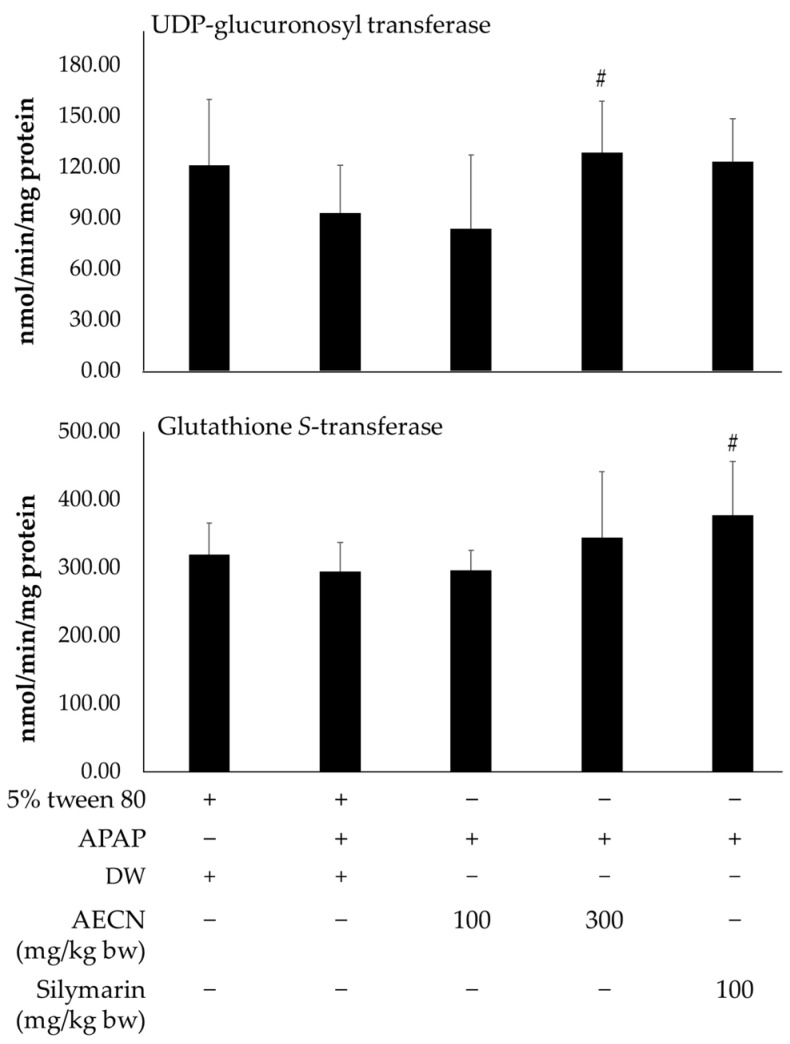
Effect of AECN on certain detoxifying enzymes in rat livers treated with APAP. Data are expressed as mean ± SD values (*n* = 8). # Significantly different from the APAP-treated group (*p* < 0.05). AECN: acidified ethanolic extract of *C. nervosum* pulp; APAP: acetaminophen at a dose of 3000 mg/kg bw; DW: distilled water used as a vehicle for AECN; 5% tween 80 used as a solvent control for APAP.

The detoxifying enzymes involved in APAP metabolism, including UDP-glucuronosyl transferase (UGT) and glutathione *S*-transferase (GST), were evaluated. The administration of APAP at this time point did not alter the activities of UGT and GST in the rat livers. Notably, the AECN treatment significantly increased the activity of hepatic UGT but not GST when compared to APAP-induced rats. The oral administration of silymarin at 100 mg/kg bw significantly modulated the GST activity in the liver of the APAP-treated group. The results of the APAP-associated metabolizing enzyme activities are demonstrated in Figure 5.

## 3. Discussion

Fruits and vegetables are considered valuable sources of beneficial compounds that are advantageous for health promotion. *Cleistocalyx nervosum* var. *paniala*, a purplish fruit, is known for a range of biological activities that include antioxidant properties, antimutagenicity, and anticarcinogenicity [18,19]. The present study has shown that the acidified ethanolic extract of *C. nervosum* pulp (AECN) contains high amounts of polyphenols that exhibit a hepatoprotective effect on overdose APAP-treated rats. The LD50 value of AECN was higher than 5000 mg/kg bw in rats, indicating its safe application as either a food or supplement.

Natural phenolic compounds, including anthocyanins, displayed a range of biological properties, particularly antioxidant activity involving the prevention of certain degenerative diseases. Various conventional and assisted extraction techniques have been used to obtain antioxidant polyphenols from plant matrices. However, both the extraction method and the solvent selection process have an impact on extraction capacity. Ethanol is a classical solvent that can be employed to force phenolic compounds from plants and has been found to be non-toxic in applications for humans [21,22]. The hydroethanolic solution could be used to extract a variety of phenolic compounds depending upon their proportions. We found the obtained phenolic contents of *C. nervosum* pulp using extraction with 80% ethanol provided a significant degree of radical scavenging activity. Moreover, the anthocyanin contents extracted under the mild acidic conditions of citric acid delivered the most effective degree of antioxidant activity of the *C. nervosum* pulp ethanolic extract. This outcome was in line with those of certain groups suggesting the conditions preventing non-acetylated anthocyanins degradation [23]. Cyanidin 3-glucoside (C3G) is a major anthocyanin in AECN. Our findings are consistent with those of some previous studies that reported that *C. nervosum* pulp contained higher amounts of C3G than other fruits [24]. Nevertheless, the content of total anthocyanins observed in AECN measured using a pH-differential method was lower than the amount of C3G detected using HPLC. The pH-differential method is a colorimetric method used to determine changes in the absorbance of the structural anthocyanin chromophore at two different pH values. The anthocyanin content was determined using the extinction coefficient and molecular weight of C3G. It was then used to express the equivalent content to C3G. HPLC analysis can be directly analyzed by comparing the specific peak area in the AECN to that of the standard anthocyanins. For these reasons, the value observed using a pH-differential method would be lower than that which had been obtained using HPLC analysis. Accordingly, this determination is in line with what has been reported in various published articles [25,26,27]. Several studies have suggested that anthocyanins could effectively ameliorate the liver injuries caused by APAP administration in rats and mice through the inhibition of lipid peroxidation, improved GSH depletion, and by inducing antioxidant enzymes. This also occurred as a result of modulating several signaling pathways that are known to be involved with hepatoprotection [17,28]. Moreover, C3G exhibited a hepatoprotective effect on hydrogen peroxide and carbon tetrachloride-induced oxidative damage via modulation of the AMPK/Nrf2 pathway [29]. Apart from C3G, chlorogenic acid was recognized as a promising phenolic acid in AECN. Accordingly, it could restrain pro-inflammatory cytokines, inhibit myeloperoxidase activity, and restore liver enzymes leading to the protection of the liver injuries induced by APAP in rats [16,30]. Furthermore, a large variety of phenolic acids and their derivatives were found in the pulp of *C. nervosum*, such as ellagic acid, gallic acid, caffeoylquinic acid, monogalloyldiglucoside, and methoxymethylgallate [31]. This outcome suggests that the hepatoprotective effect of AECN can be elaborated with various phytochemicals in *C. nervosum.*

Hepatotoxicity includes either liver injuries or liver failure that has been triggered by exposure to particular natural or environmental toxicants and/or an overdose of certain drugs. APAP is an analgesic and antipyretic drug that has been determined to be safe at a therapeutic dose, while it can cause significant clinical hepatotoxicity at a very high dose. Lipid peroxidation caused by a hydroxyl radical interaction has been found to alter the membrane potential transition leading to ATP depletion and necrotic cell death, which is considered a key event in APAP toxicity [7,32]. Our findings reveal that the administration of an overdose of APAP perturbed the hepatic oxidative balance, leading to liver dysfunction and morphological changes in rats. Interestingly, the administration of AECN could alleviate the hepatotoxic effect of APAP supported by decreases in the serum ALT and MDA levels and the recovered hepatocyte architecture. Numerous natural products have demonstrated the hepatoprotective capacity on APAP-induced liver injuries via different mechanisms [33]. The reduction in cellular free radical production is one strategy that could lessen oxidative source-induced toxicity. The treatment of AECN at 100 mg/kg bw for 28 days diminished lipid peroxidation in the serum, while the administration of AECN at 300 mg/kg bw of silymarin could decrease the effect in both serum and liver samples. Therefore, it can be suggested that the functional ingredients in AECN, particularly C3G, might play a role in radical-scavenging capacities. This result is consistent with the previously reported outcomes on in vitro DPPH activity, for which AECN was found to contain high anthocyanin content and to possess significant radical scavenging capabilities. Furthermore, the antioxidant system is another crucial strategy in regulating cellular redox homeostasis. GSH plays a significant role in GSH-related enzymes consisting of GPx, GR, and GST. Accordingly, GPx converts H_2_O_2_ into water, while GSH is transformed to oxidized glutathione (GSSG), which is then further recycled to its reduced form by GR [1]. In this study, AECN restored GSH and increased the activity of GPx in rat livers, whilst silymarin, a well-known herbal hepatoprotectant, statistically augmented GSH recycling enzyme activities including GPx, GR, and GST. These defense mechanisms of AECN might involve a protective effect on oxidative stress-induced liver injuries. Nuclear factor erythroid 2-related factor 2 (Nrf2) is an oxidant stress response transcription factor that regulates macromolecule metabolism, inflammation, autophagy, and immune responses. The binding of Nrf2 occurs via an antioxidant response element targeted on the transcription of several antioxidant enzymes and by detoxifying enzymes to prevent hepatopathogenesis [34,35,36]. Some phytochemical antioxidants enhanced the Nrf2/ARE signaling pathway, resulting in the activation of their downstream target proteins [37,38]. AECN could effectively elevate the activities of antioxidant enzymes through an enhanced transcriptional activation of the Nrf2 signaling pathway, leading to a lowering of APAP-initiated oxidative stress in rat livers. Notably, the increased level of GSH content was higher than that which was associated with GPx and GR activities. It has been implied that some phenolic compounds in AECN might be able to modify, not only antioxidant enzymatic system, but also the GSH synthesis pathways, such as those associated with glutamylcysteine synthetase and GSH synthase [39,40].

Detoxifying systems that particularly involve phase II xenobiotic metabolizing enzymes via a conjugation reaction have been recognized as an important cellular defense mechanism on toxicants [41]. UGT and SULT are primarily phase II enzymes in APAP detoxification. When these enzymes are insufficient, excessive APAP will be metabolized by hepatic microsomal cytochrome P450 2E1 generating a highly reactive N–acetyl–p–benzoquinamine (NAPQI) metabolite, resulting in liver malfunction. However, NAPQI will be further eliminated by GST through conjugation with GSH [4,5,7]. This study found that UGT activity was markedly increased in high doses of AECN administration, whereas silymarin treatment could significantly enhance GST activity to ameliorate the oxidative stress induced by APAP. Importantly, the mechanism underlying AECN and silymarin against APAP-induced acute hepatotoxicity may be different.

## 4. Materials and Methods

### 4.1. Chemicals

4-Nitrophenol, 5,5′-dithiobis-2-nitrobenzoic acid (DTNB), APAP, GR, GSH and oxidized glutathione (GSSG), thiobarbituric acid (TBA), tert-butyl hydroperoxide (t-BHP), and all standard phenolic acids were purchased from Sigma Aldrich Corp. (St. Louis, MO, USA). Furthermore, 1-Chloro-2,4-dinitrobenzene (CDNB) was provided by Thermo Fisher Scientific Inc. (Waltham, MA, USA), while β-NADPH was purchased from Nacalai Tesque (Kyoto, Japan). Trichloroacetic acid (TCA) and H_2_O_2_ were obtained from Merck Millipore (Burlington, MA, USA). All standard flavonoids and anthocyanins were obtained from Sigma Aldrich Corp. (St. Louis, MO, USA) and Extrasynthese (Genay, France). HPLC grades of water, methanol, and acetonitrile were obtained from RCI labscan Ltd. (Bangkok, Thailand).

### 4.2. Preparation of C. nervosum Extract

Fruit specimens of *C. nervosum* were collected from the Horticulture Research Center Region 1, Office of Agricultural Research and Development, Department of Agriculture, Thailand. *C. nervosum* voucher numbers QGB 7290, QGB 17340, and QGB 25139 were deposited at the Queen Sirikit Botanical Garden, Chiang Mai, Thailand. The flesh was manually removed from seeds of the fruit, and the flesh were twice extracted with distilled water or 20, 40, 60, 80, and 95% (*v*/*v*) ethanol and acidified ethanol (0.5% (*v*/*v*) citric acid in 80% (*v*/*v*) ethanol) for 24 h. They were subsequently centrifuged at 3000 rpm for 15 min. The extracts were then evaporated and lyophilized in order to obtain *C. nervosum* (CN) pulp extracts. The crude extracts were then stored at −20 °C until being used.

### 4.3. Analysis of Phytochemical Contents Using Spectrophotometry

The total phenolic compound and flavonoid content in each extract was determined using the Folin–Ciocalteu method and aluminum chloride colorimetric method, respectively, as has been described by Sankam et al. [42]. Gallic acid was representative of a standard polyphenol, while catechin was used as a standard flavonoid. The total phenolic content was expressed as mg of gallic acid equivalent (GAE) per 100 g of extract. The total flavonoid content was expressed as mg of catechin equivalent (CE) per 100 g of extract.

Total anthocyanin content was determined using the pH-differential method, as has been described by Lee et al. [43], with slight modifications. A sample with an appropriate dilution was added to 0.025 M potassium chloride buffer at a pH value of 1.0 and 0.4 M sodium acetate buffer at a pH value of 4.5. The absorbance of each mixture was measured at 510 and 700 nm, respectively. Anthocyanin content was calculated according to the difference in absorbance values between pH 1.0 and pH 4.5 and expressed as mg of cyanidin-3-glucoside (C3G) equivalents per g of extract.

### 4.4. High Performance Liquid Chromatography for Antioxidant Fraction of CN Pulp

Reverse-phase high performance liquid chromatography (HPLC) was conducted to measure phenolic acids, and anthocyanins in the acidified ethanol extract of CN pulp (AECN). Assay conditions were performed on a Zorbax Eclipse Plus C18 column (Agilent 4.6 mm × 250 mm, 5 µm) and analyzed using an Agilent HPLC 1260 infinity series system (Agilent, Santa Clara, CA, USA) at a constant flow rate of 1 mL/min and 10 uL of the injection volume.

An examination of phenolic acids in AECN was performed as has been described elsewhere [44]. The mobile phase consisted of 3% acetic acid in water (A) and methanol (B). The gradient elution was initiated with 90% of A and then changed to 85 and 70% of eluent A at 15 and 40 min, respectively. Wavelengths at 260, 280, and 320 nm were used to measure a range of phenolic acids. Gallic acid, protocatechuic acid, 4-hydroxybenzoic acid, chlorogenic acid, vanillic acid, syringic acid, and *p*-coumaric acid were used as the standard phenolic acids.

The determination of anthocyanins in AECN was performed according to the method described by Shim et al. [45] with slight modifications. Anthocyanins were detected at a wavelength of 520 nm using a gradient system of 5% formic acid in water (A) and acetonitrile (B) initiated from 100% A to 85% A within 15 min and was subsequently returned to 100% A within 5 min. Anthocyanins were measured at 520 nm. The anthocyanin standards were cyanidin-3-glucoside (C3G), delphinidin-3-glucoside (D3G), malvidin-3-glucoside (M3G), and peonidin-3-glucoside (P3G).

### 4.5. In Vitro Antioxidant Activity of CN Pulp Extracts

The free radical scavenging activity of CN extract was investigated according to the method described by Lengkidworraphiphat et al. [46]. Various concentrations of the extract were added to 0.2 mM 2,2-diphenyl-1-picrylhydrazyl (DPPH) reagent and incubated in the dark at room temperature for 30 min. The resulting concentration was monitored at a wavelength of 517 nm. Ascorbic acid was used as a positive control. The percentage inhibition was plotted against various concentrations of CN extracts, while the linear equation was used to calculate the IC_50_ value. The IC_50_ value of an antioxidant is the concentration at which 50% of free radical activity is inhibited.

### 4.6. Animals and Experimental Design

All rats were obtained from the National Laboratory Animal Center, Mahidol University, Nakhon Pathom, Thailand. They were housed under controlled conditions of 25 °C and a 12-hour dark–light cycle, while being allowed free access to water and diet. The experimental protocols were approved of by the Animal Ethics Committee of the Faculty of Medicine, Chiang Mai University (Approval number: 26/2564). 

Acute toxicity test of AECN was performed by following the guidelines established by the Organization for Economic Co-operation and Development (OECD) number 425 [47]. Ten female Wistar rats (8 weeks old and approximately 200 g) were divided into 2 groups of 5 rats each. The control group orally received 4 mL/kg bw of water, while the treated group was orally fed 5000 mg/kg bw of AECN. On day 15, all rats were sacrificed and their internal organs, including the heart, lungs, thymus, liver, pancreas, adrenal glands, spleen, kidneys, stomach, ovaries, and fallopian tubes, were weighed and observed.

The hepatoprotective effect of AECN was subsequently determined. Forty rats were divided into 5 groups. Group 1 received 4 mL/kg bw of distilled water and fed with 5% tween 80 on day 28 of the experiment and served as a negative control group. The rats in group 2, a positive control group, were fed with 4 mL/kg bw of distilled water and administered with APAP at a dose of 3000 mg/kg bw on day 28 of the experiment. AECN was orally administered to rats in groups 3–4 at dosages of 100 and 300 mg/kg bw. Group 5 was given silymarin at 100 mg/kg. On day 28, after 1 h of intragastric feeding of the extracts, rats were intragastrically administrated with 3000 mg/kg bw of APAP. After 24 h of APAP treatment, all rats were sacrificed, and their blood was collected for ALT analysis. The liver, spleen, and kidneys were removed and weighed. Livers were cut into three pieces and fixed in 10% phosphate buffered formalin for histopathological analysis using hematoxylin and eosin (H&E) staining. The liver sections were evaluated for pathological signs of hepatotoxicity such as necrosis, fatty infiltration, fibrosis, and lymphocyte infiltration. The remaining liver samples were stored at −80 °C for further biochemical analysis.

### 4.7. Preparation of Liver Cytosolic and Microsomal Fraction

The liver tissue samples were homogenized and centrifuged at 10,000 rpm for 20 min at 4 °C and ultracentrifuged at 100,000× *g* for 60 min at 4 °C, as has been previously described by Phannasorn et al. [48]. The cytosolic supernatant and microsomal pellets were obtained and stored at −80 °C for further analysis. The total protein concentration of each sample was determined using the Lowry method and reported as mg protein/mL using a BSA calibration curve.

### 4.8. Oxidative Stress Markers in Serum and Liver Tissue

Lipid peroxidation in blood serum and liver tissue samples was investigated using thiobarbituric acid reactive substance (TBARS) assay according to certain specific publications [48,49]. Serum was mixed with 0.2% butylated hydroxytoluene (BHT) and 0.44 M H_3_PO_4_ and incubated at room temperature for 10 min. A solution comprised of 0.6% thiobarbituric acid (TBA) was added and then boiled for 30 min. The resultant solution was spectrophotometrically monitored at 540 nm after centrifugation at 12,000 rpm for 5 min. The amount of malondialdehyde (MDA), the end-product in the TBARS assay, was quantified and used as an index of lipid peroxidation.

The liver homogenate was precipitated in 50% trichloroacetic acid (TCA) and centrifuged at 6000 rpm, 4 °C for 20 min. TBA was added to the supernatant and the mixture was heated to 100 °C for 10 min. The reactions were stopped by being placed on ice. Butanol was then added, and the reactions were centrifuged at 3000 rpm for 10 min and were then monitored at 532 mm. 

### 4.9. Glutathione Content in Serum and Liver Tissue

The content of glutathione present in the blood serum and liver samples was determined using the GSH recycling system, as has been previously described [48]. Deproteinized serum and liver specimens were mixed with a reaction mixture containing 10 mM sodium phosphate buffer in conjunction with 5 mM EDTA (pH 7.5), 4 mM β-NADPH, 6 U GR, and 10 mM DTNB. The resultant solution was spectrophotometrically measured at a wavelength of 405 nm after 30 min of incubation. Using a calibration curve, the glutathione concentration in the samples was calculated and expressed as nmol/mg protein.

### 4.10. Antioxidant Enzyme Activities in Rat Liver

In order to determine CAT activity, the cytosolic fraction was mixed with a reaction mixture containing 30 mM H_2_O_2_ and 50 mM phosphate buffer (pH 7.0). Catalase activity was estimated by a decrease in the absorbance of H_2_O_2_ at 240 nm. CAT activity was then calculated and expressed as µmol of H_2_O_2_/min/mg protein [19,48].

GPx activity was determined by adding cytosol to a reaction mixture comprised of 0.1 M Tris-EDTA buffer (pH 8.0), 0.1 M GSH, 2 mM β-NADPH, 7 mM t-BHP, and 10 U GR. The oxidation of β-NADPH was monitored at a wavelength of 340 nm and expressed as µmol/min/mg protein [19,48].

To determine GR activity, a reaction mixture containing 100 mM potassium phosphate buffer (pH 7.0), 1.2 mM GSSG, and 1.2 mM β-NADPH was employed. The degree of reduction in the absorbance of β-NADPH was spectrophotometrically monitored at 340 nm. The GR activity was reported as µmol/min/mg protein [19,48].

### 4.11. Xenobiotic Metabolizing Enzyme Activities

GST activity was examined according to Chariyakornkul et al. [44]. Briefly, a reaction mixture comprised of 0.2 M potassium phosphate buffer (pH 6.5), 10 mM GSH, deionized water, and diluted cytosol was employed. The substrate was CDNB and measured at 340 nm. The enzyme activity was quantified as µmol CDNB conjugate formed/min/mg protein using a molar coefficient of 9.6 M^−1^ cm^−1^.

The activity of UGT was determined according to the method described by Chariyakornkul et al. [44] using 4-NP as a substrate. UDP-glucuronic acid was added to the reaction mixture containing diluted microsomal fraction, 100 mM Tris-HCl (pH 8.5), 4 mM MgCl_2_, and 0.5 mM 4-NP. It was then incubated at 37 °C for 20 min. This reaction was halted by adding 10% TCA and the supernatant was then collected by centrifuging the reaction at 10,000× *g* for 15 min. The supernatant was alkalinized with 0.5 M NaOH and measured at 405 nm. Enzyme activity was expressed as nmol of 4-NP conjugate formed/min/mg protein.

### 4.12. Data Analysis

All data are reported as mean ± SD values. All experiments were conducted independently at least three times. The statistical significance of the differences between all groups was determined using one-way analysis of variance (ANOVA) and the post hoc least-significant difference (LSD) or Duncan Multiple Range test. A *p*-value of less than 0.05 was regarded as being significant.

## 5. Conclusions

AECN was found to be rich in anthocyanins and safe for administration in rats due to its LD50 value. It effectively ameliorated hepatotoxicity in overdose APAP-induced rats through the induction of certain antioxidant enzymes and detoxifying enzymes leading to the restoration of GSH and a reduction in oxidative stress. Consequently, *C. nervosum* pulp may offer new insights as a functional ingredient for effective health promotion.

## Figures and Tables

**Table 1:** Phytochemical constituents and IC_50_ values of scavenging activity using DPPH assay in various extracts of CN pulp.

Extracts	Total Phenolic Compounds (mg eq GAE/g Extract)	Total Flavonoid Compounds (mg eq CE/g Extract)	Total Anthocyanin (mg eq C3G/g Extract)	IC_50_ Value of DPPH Radical Scavenging Activity (mg/mL)
Aqueous Extract	13.50 ± 1.55 ^b^	2.38 ± 0.55 ^f^	0.733 ± 0.026 ^e^	9.58 ± 0.42 ^e^
20% EtOH Extract	15.78 ± 0.94 ^b^	2.95 ± 0.75 ^f^	1.098 ± 0.014 ^e^	7.45 ± 0.16 ^d^
40% EtOH Extract	22.64 ± 3.16 ^b^	4.16 ± 0.87 ^e^	4.176 ± 0.336 ^c^	5.89 ± 0.09 ^c^
60% EtOH Extract	40.82 ± 6.30 ^a^	6.84 ± 0.35 ^d^	3.569 ± 0.316 ^d^	4.51 ± 0.66 ^b^
80% EtOH Extract	45.17 ± 3.62 ^a^	11.36 ± 0.10 ^b^	5.889 ± 0.086 ^b^	4.43 ± 0.25 ^b^
95% EtOH Extract	40.76 ± 4.64 ^a^	8.82 ± 0.72 ^c^	4.257 ± 0.159 ^c^	4.88 ± 0.02 ^b^
0.5% Citric Acid in 80% EtOH Extract	50.47 ± 4.67 ^a^	13.85 ± 0.63 ^a^	7.282 ± 0.025 ^a^	3.25 ± 0.27 ^a^

Data are expressed as mean ± SD values of three independent experiments; ^a–f^ Values presented in different letters within the same column differ significantly (*p* < 0.05); IC_50_ value of DPPH radical scavenging activity of ascorbic acid (standard antioxidant) is 0.18 ± 0.08 mg/mL; GAE: gallic acid equivalent, CE: catechin equivalent, C3G: cyanidin-3-glucoside.

**Table 2:** Effect of AECN on certain oxidative stress markers in the serum and livers of APAP-treated rats.

Groups	Serum		Liver	
	MDA (pmol/mg Protein)	GSH (nmol/mg Protein)	MDA (pmol/mg Protein)	GSH (nmol/mg Protein)
DW + 5% Tween-80	59.05 ± 11.92	28.36 ± 6.65	60.92 ± 26.11	18.10 ± 1.28
DW + APAP	74.74 ± 7.97 *	29.67 ± 6.64	84.96 ± 21.05 *	5.25 ± 0.91 *
AECN 100 mg/kg bw + APAP	51.34 ± 4.60 ^#^	27.21 ± 4.98	65.16 ± 25.74	6.66 ± 4.11
AECN 300 mg/kg bw + APAP	52.21 ± 3.27 ^#^	29.86 ± 3.89	46.65 ± 18.32 ^#^	12.41 ± 2.00 ^#^
Silymarin 100 mg/kg bw + APAP	49.85 ± 11.31 ^#^	31.42 ± 4.63	49.18 ± 20.83 ^#^	2.69 ± 0.98

Data are expressed as mean ± SD (*n* = 8); * Significantly different from the control group (*p* < 0.05), ^#^ Significantly different from the APAP-treated group (*p* < 0.05); AECN: acidified ethanolic extract of *C. nervosum* pulp, APAP: acetaminophen at dose 3000 mg/kg bw, ALT: alanine aminotransferase, DW: distilled water, MDA: malondialdehyde, GSH: glutathione, DW: distilled water used as a solvent control for AECN; 5% tween 80 used as a solvent control for APAP.

## Data Availability

The data presented in this study are available on request from the corresponding author.

## Sample Availability

Samples of the compounds are available from the authors.
